# Supplementary material for: A choanoflagellate cGLR-STING pathway reveals evolutionary links between bacterial and animal immunity
Source: bioRxiv. 2025 Sep 4:2025.09.04.674280. Preprint. [Version 1] doi: 10.1101/2025.09.04.674280 (PMC12424679; doi:10.1101/2025.09.04.674280)
Supplement: Supplement 1 [file media-1.pdf]

Supplementary Table 2. Crystallographic Statistics, related to Fig. 3

	*Sm*STING–2′3′-cGAMP (SeMet)	*Sm*STING–2′3′-cGAMP
**Data Collection**		
Resolution (Å)[a]	75.90–3.32 (3.50–3.32)	34.04–2.65 (2.71–2.65)
Wavelength (Å)	0.9792	0.92010
Space group	$P\ 1\ 2_1\ 2$	$P\ 1\ 2_1\ 2$
Unit cell: a, b, c (Å)	66.39, 88.30, 76.15	66.95, 88.74, 76.02
Unit cell: α, β, γ (°)	90.0, 94.6, 90.0	90.0, 94.1, 90.0
Molecules per ASU	2	2
Total reflections	82710 (8528)	184837 (13747)
Unique reflections	12067 (1379)	25932 (1855)
Completeness (%)[a]	92.2 (72.9)	99.7 (96.8)
Multiplicity[a]	6.9 (6.2)	7.1 (7.4)
$I/\sigma I$[a]	5.4 (0.6)	4.51 (0.77)
CC(1/2)[b] (%)[a]	99.2 (38.4)	98.4 (37.8)
Rpim[c] (%)[a]	11.2 (155.2)	11.3 (84.6)
Sites	20	
**Refinement**		
Resolution (Å)		34.04–2.65
Free reflections		2024 (145)
R-factor / R-free		24.3 / 27.5
Bond distance (RMS Å)		0.003
Bond angles (RMS °)		0.63
**Structure/Stereochemistry**		
No. atoms: protein		5759
No. atoms: ligand		45
No. atoms: solvent		79
Average B-factor: protein		71.96
Average B-factor: ligand		60.80
Average B-factor: water		56.81
Ramachandran plot: favored		98.04%
Ramachandran plot: allowed		1.96%
Ramachandran plot: outliers		0.00%
Rotamer outliers		0.65%
MolProbity[d] score		1.46
Protein Data Bank ID		9Q1F

[a] Highest resolution shell values in parenthesis
[b] (Karplus and Diederichs, 2012)
[c] (Weiss, 2001)
[d] (Chen et al., 2010)
